# What’s in your next-generation sequence data? An exploration of unmapped DNA and RNA sequence reads from the bovine reference individual

**DOI:** 10.1186/s12864-015-2313-7

**Published:** 2015-12-29

**Authors:** Lynsey K. Whitacre, Polyana C. Tizioto, JaeWoo Kim, Tad S. Sonstegard, Steven G. Schroeder, Leeson J. Alexander, Juan F. Medrano, Robert D. Schnabel, Jeremy F. Taylor, Jared E. Decker

**Affiliations:** Informatics Institute, University of Missouri, Columbia, MO 65211 USA; Division of Animal Sciences, University of Missouri, Columbia, MO 65211 USA; Embrapa Southeast Livestock, São Carlos, São Paulo 13560-970 Brazil; Animal Genomics and Improvement Laboratory, USDA-ARS, Beltsville, MD 20705 USA; Recombinetics Inc., 1246 University Ave W #301, St Paul, MN 55104 USA; USDA-ARS (retired), LARRL, Fort Keogh Miles City, Montana, 59301 USA; Department of Animal Science, University of California-Davis, Davis, CA 95616 USA

**Keywords:** DNA sequencing, RNA sequencing, Unmapped reads

## Abstract

**Background:**

Next-generation sequencing projects commonly commence by aligning reads to a reference genome assembly. While improvements in alignment algorithms and computational hardware have greatly enhanced the efficiency and accuracy of alignments, a significant percentage of reads often remain unmapped.

**Results:**

We generated *de novo* assemblies of unmapped reads from the DNA and RNA sequencing of the *Bos taurus* reference individual and identified the closest matching sequence to each contig by alignment to the NCBI non-redundant nucleotide database using BLAST. As expected, many of these contigs represent vertebrate sequence that is absent, incomplete, or misassembled in the UMD3.1 reference assembly. However, numerous additional contigs represent invertebrate species. Most prominent were several species of Spirurid nematodes and a blood-borne parasite, *Babesia bigemina*. These species are either not present in the US or are not known to infect taurine cattle and the reference animal appears to have been host to unsequenced sister species.

**Conclusions:**

We demonstrate the importance of exploring unmapped reads to ascertain sequences that are either absent or misassembled in the reference assembly and for detecting sequences indicative of parasitic or commensal organisms.

**Electronic supplementary material:**

The online version of this article (doi:10.1186/s12864-015-2313-7) contains supplementary material, which is available to authorized users.

## Background

Next-generation sequencing technology has vastly increased the dimensionality of sequencing projects and routinely allows the generation of hundreds of millions or even billions of short reads. Analysis of these data requires that the short reads be assembled into contiguous sequences either using *de novo* or reference-guided assembly. For organisms with a reference genome, reads generated in the sequencing process are usually matched to the reference sequence with a variety of alignment algorithms. This is currently the most efficient way of transforming the raw sequence reads into a consensus sequence. However, there are several limitations inherent to the alignment process, including alignment to repetitive regions, absent or misassembled sequence in the reference genome, and individual genetic divergence between the subject organism’s genome and the reference genome [1]. Despite these challenges, the majority of reads produced from a sequencing experiment will adequately align to a reference assembly. Nevertheless, a small but significant fraction of reads frequently remain unmapped.

Unmapped reads have generally been disregarded and these data are often discarded. However, recent work has begun to focus on the development of bioinformatic tools for detecting pathogens in human sequence data by the computational subtraction of known human sequences [2–4]. Application of these pipelines in other recent studies has suggested that potentially biologically relevant information can be extracted from the unmapped reads [5, 6]. Using an original alignment, assembly, and identification pipeline that can be applied to data from any species, we took advantage of a unique opportunity to explore the unmapped reads from the DNA and RNA sequencing of L1 Dominette 01449, the *Bos taurus* reference individual [7]. These data had not previously been used in the creation or annotation of the reference assembly.

Using sequence data produced from the reference individual, we minimized alignment challenges that are due to genetic variation among individuals. Thus, we expected to encounter meaningful biological information pertaining to sequences poorly represented in the bovine reference assembly and sequences indicative of parasitic or commensal non-vertebrate organisms. We identified DNA and RNA contigs that were assembled *de novo* from unmapped reads that could generally be classified into one of three categories: 1) sequence from bovine; 2) sequence from other vertebrate species that was homologous to bovine; and 3) sequence from non-vertebrate species. This analysis unequivocally demonstrates that the unmapped reads contain important data pertaining to sequences from the organism that are missing from the reference assembly, represented by categories 1 and 2, and sequences that can be used to identify microbiota members, putatively represented by category 3.

## Results

### De novo assembly of unmapped reads

Approximately 111.7 million DNA sequence reads, 7.2 % of the total, remained unmapped after alignment to the reference genome. A fraction of those reads could be used for assembly, due to a large number of sequences with low quality (Additional file 1: Table S1). However, approximately 1.4 million reads were incorporated into 69,230 contigs with an N50 of 737 bp. Overall, the contigs comprised approximately 46.6 Mb. Additional assembly statistics are provided in Additional file 1: Table S1.

A median of approximately 6.7 % of RNA-seq reads remained unmapped across each of the 17 tissue samples. *De novo* assembly of these reads yielded a total of 43,961 contigs, with a median of 1792 contigs per tissue and an N50 of 324.5 bp. Overall, the contigs spanned 14.8 Mb with a median of 603 Kb per tissue. Assembly statistics for each tissue are in Additional file 1: Table S2.

### Pairwise alignment of contigs assembled from unmapped DNA reads to the non-redundant nucleotide database

**Fig. 1:**
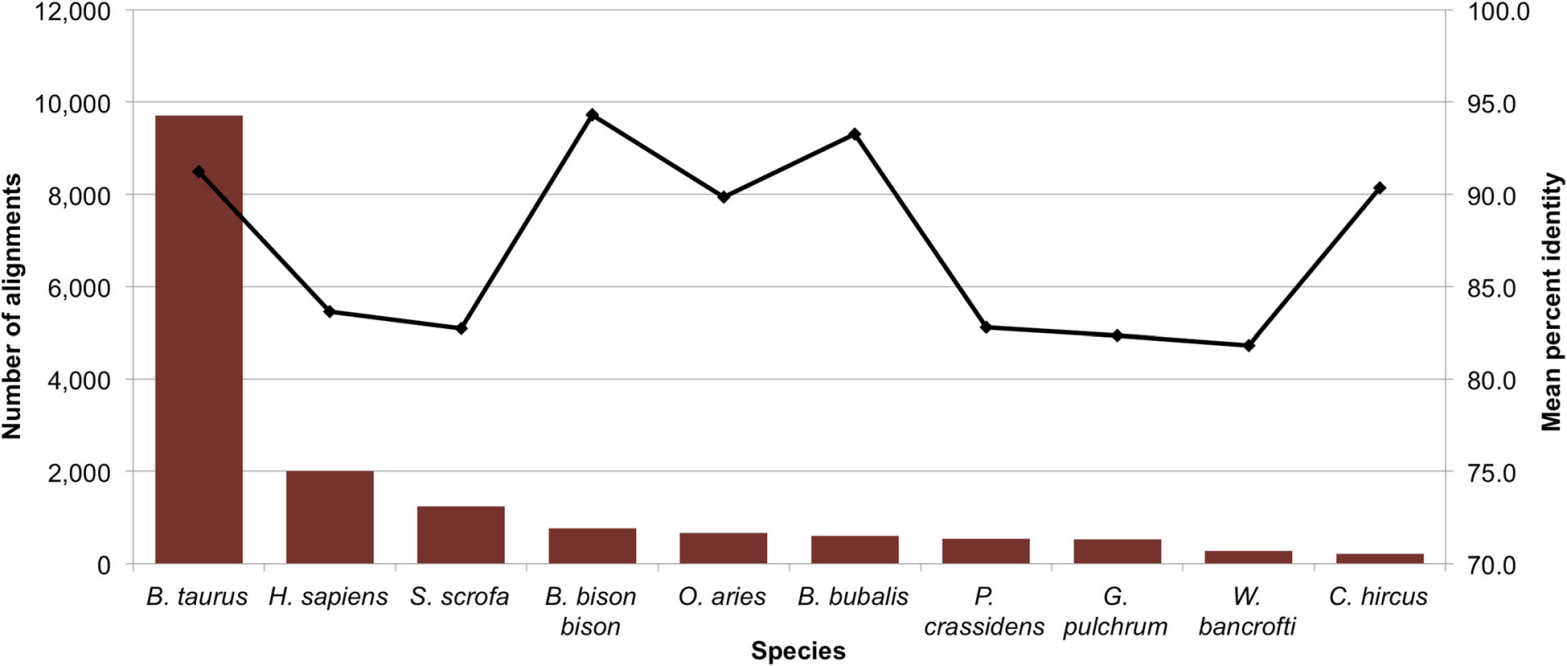
Most common alignments from DNA. Bar chart of the ten most common species with significant alignments from the pairwise alignment of *de novo* assembled contigs from unmapped DNA reads. Trend line represents the median percent identity for each species

Approximately 51 % of the contigs generated from the unmapped DNA reads produced a significant alignment when queried against the non-redundant nucleotide (*nt*) database. The most common alignment was to other *Bos taurus* sequences (Fig. 1). This result was expected given the draft quality of the bovine reference assembly and considering that we assembled paired reads if either one or both of the reads were unmapped to the reference assembly. However, the second most common alignment for these DNA contigs was to *Onchocerca ochengi*, a nematode known to infect indicine cattle that has been heavily researched due to its similarity to the parasite that causes African River Blindness in humans. We simulated paired-end sequence read data from the *O. ochengi* genome assembly by randomly shearing the genome and then aligned the produced paired-end reads to the bovine reference assembly and concluded that the *O. ochengi* assembly is contaminated with cattle sequences (Additional file 2: Note 1). Consequently, we excluded the *O. ochengi* assembly from any further analyses.

With subsequent analyses preventing alignment to *O. ochengi*, approximately 44 % of the contigs produce a significant alignment against the *nt* database. A fraction of the contigs originally identified as *O. ochengi* were unambiguously matched to bovine sequences. However, the number of alignments to other filarial nematode sequences also increased. These included hundreds of contigs aligned to *Gonglyonema pulchrum* and *Wuchereria bancrofti,* and a few to *Parascaris equorum. G. pulchrum* and *W. bancrofti* belong to the order Spirurida, as does *O. ochengi*, but that are known to only infect humans. The alignments to each of these species had a percent identity of approximately 82 % (Table 1), which is consistent with cattle not being a host for these nematodes and indicating that these alignments represent sequences from unsequenced sister species of *G. pulchrum* and *W. bancrofti*.

**Table 1:** Top four non-vertebrate alignments to *de novo* assembled contigs from unmapped DNA sequence reads

Species	Number of Alignments	Median Identity (%)	Maximum Identity (%)	Median Length (bp)	Maximum Length (bp)	Median E-Value
*Gongylonema pulchrum*	516	82.33	100	641.0	1,008	2.00E-134
*Wuchereria bancrofti*	273	81.35	98.82	640.0	1,607	1.53E-143
*Babesia bigemina*	206	91.10	100.00	505.0	2,078	3.50E-179
*Parascaris equorum*	11	81.17	100	206.0	1,008	4.00E-41

**Table 2:** Top four non-vertebrate alignments to *de novo* assembled contigs from unmapped RNA-seq reads

Species	Number of Alignments	Median Identity (%)	MaximumIdentity (%)	Median Length (bp)	Maximum Length (bp)	Median E-Value
*Uncultured bacterium*	34	97.11	100.00	274.0	1,381	2.01E-116
*Bovine herpesvirus 6*	22	99.18	100.00	294.5	933	1.00E-143
*Onchocerca flexuosa*	13	87.74	89.12	224.0	510	3.00E-57
*Babesia bigemina*	12	94.35	99.51	379.5	926	1.00E-151

Also detected were sequences with high percent identities to *Babesia bigemina,* a blood-borne parasite known to cause bovine babesiosis, or Texas fever in cattle. While only 190 contigs aligned to *B. bigemina*, significantly less than the combined number of alignments to nematode species, ten were larger than 1000 bp and the median identity was 91.10 % (Table 1). A complete summary of significant alignments, both vertebrate and non-vertebrate, is presented in Additional file 1: Table S3.

Alignments to other vertebrates represent cattle sequences that are not currently well represented in the *Bos taurus* database. Thus, the number of alignments to these organisms is a function of the completeness of the available data for each species and the phylogenetic relationship between the species and cattle. For example, human (*Homo sapiens*), being the most complete, has the largest number of alignments of the other vertebrate species, followed by pig (*Sus scrofa*), and while bison (*Bison bison bison*) and water buffalo (*Bubalus bubalis*) are more closely related to cattle than human or pig, these bovids have less sequence data available and thus do not produce as many alignments.

### Pairwise alignment of contigs assembled from unmapped RNA-seq reads to the non-redundant nucleotide database

**Fig. 2:**
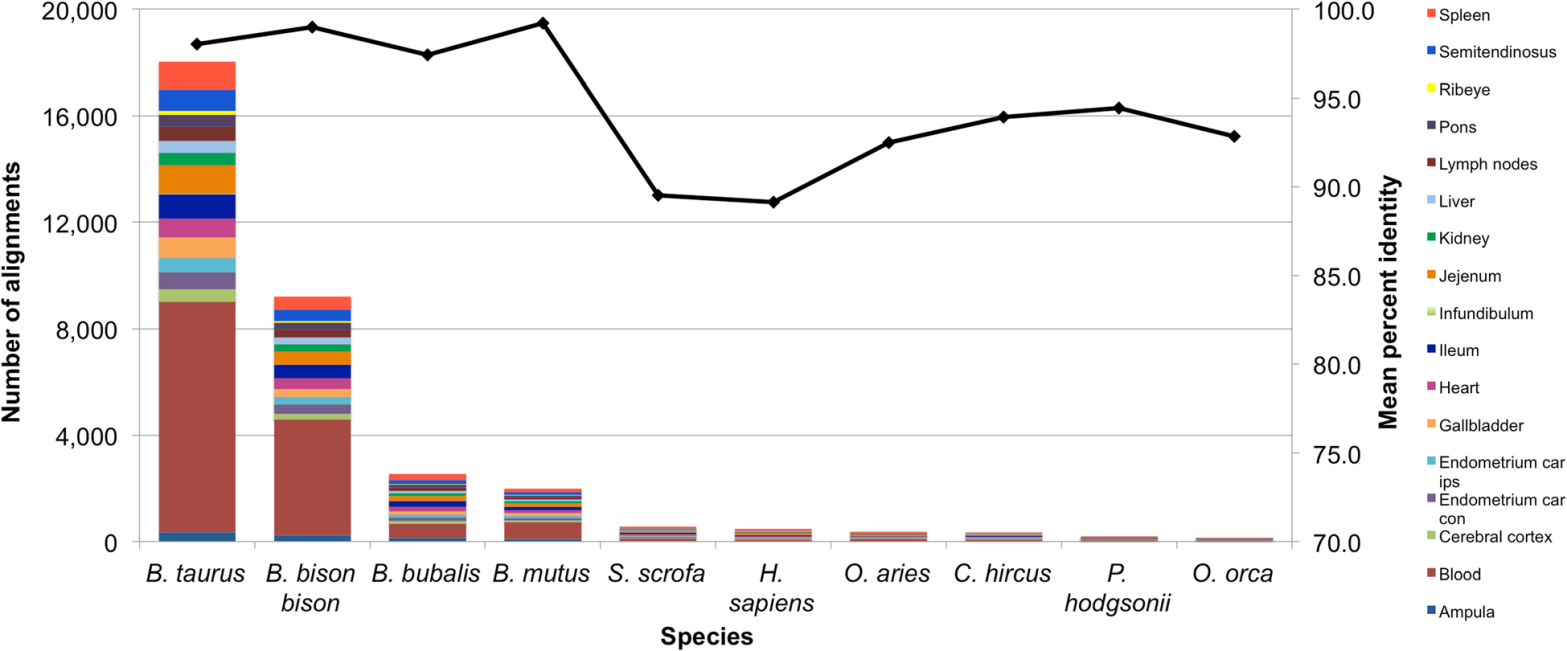
Most common alignments from RNA. Bar chart of the ten most common species with significant alignments from the pairwise alignment of *de novo* assembled contigs from unmapped RNA-seq reads by tissue. Trend line represents the overall median percent identity for each species across tissues

The pairwise alignment of the *de novo* assembled contigs generated from the unmapped RNA-seq reads to the *nt* database produced similar results to the alignment of the DNA contigs. Overall, 81 % of the RNA-seq contigs had significant alignments to sequences in the *nt* database. Across all tissues, *Bos taurus* produced the largest number of alignments. Also prominent were alignments to *Bison bison bison, Bubalus bubalis,* and *Bos mutus*, all species that are closely related to cattle (Fig. 2). Significant BLAST alignments of the RNA-seq unmapped read contigs to cattle or these other closely related species indicates the existence of coding regions that are missing or misassembled in the reference assembly. By mapping the GI number of the most significant BLAST alignment to a gene symbol, we detected alignments to 4412 *B. taurus* and 4029 *B. bison bison, B. bubalis*, or *B. mutus* genes. As the total number of *Bos taurus* genes reported by Ensembl is 19,994 [8], this suggests that as many as 42.2 % of the bovine protein coding genes are misassembled (although these misassemblies likely represent a small fraction of total transcriptome base pairs). Additionally, approximately 5 % of RNA alignments failed to map to a gene with an assigned symbol, likely corresponding to unannotated structural or regulatory RNAs. Further results and discussion of these analyses are included in Additional file 2: Note 2.

As was the case for the pairwise alignment of unmapped DNA contigs, there were also numerous alignments to other vertebrate and non-vertebrate species. The most common alignments to non-vertebrate species included uncultured bacterium, bovine herpesvirus 6, *Onchocerca flexuosa* and *B. bigemina* (Table 2). Bovine herpesvirus 6 was previously discovered as a contaminant in the UMD3.1 build by Merchant *et al*. [9], who concluded that Dominette must have been host to the virus. Alignments to *O. flexuosa* and *B. bigemina* support the hypothesis generated from the analysis of the unmapped DNA read contigs that Dominette was also host to a nematode of the Spirurida order and an unsequenced relative of *B. bigemina*. Several additional fungal and bacterial species were also detected in the unmapped read RNA-seq contigs at low levels. Nearly all of the detected non-vertebrate organisms had alignments from multiple tissues, which would be expected for blood-borne parasites. The number of alignments for each tissue was a function of the total number of sequencing reads from that tissue. A complete summary of alignments from all 17 tissues is presented in Additional file 1: Tables S4 and S5.

### No evidence of horizontal gene transfer

With deep sequencing, it is possible to expose rare horizontal gene transfer events. To address this possibility, we searched for mate-pair reads from the large insert DNA libraries where one mate was uniquely mapped to the cattle reference genome and the other mate mapped uniquely to a non-vertebrate sequence. No such mate-pairs were identified that met these criteria. Additionally, in our BLAST results we also searched for chimeric contigs (contigs that partially mapped uniquely to cattle and partially mapped uniquely to a non-vertebrate species). Again, no such contigs were identified that met these criteria.

## Discussion

To our knowledge, this is the first formal investigation into the nature and identity of unmapped reads from the resequencing of an individual used for the generation of a reference genome assembly. These data allowed us to directly compare reads to the reference assembly without alignment challenges due to genetic variation between the reference and the resequenced genome. Second, the opportunity to compare independently generated datasets from the same individual provided unequivocal support for our discovery of concordant non-vertebrate sequences within the whole genome and transcriptome sequences of the bovine host. In addition to our sequencing of cDNA generated from RNA isolated from 17 tissues, we also sequenced genomic DNA that had been isolated from both liver and white blood cells at three separate facilities. Endogenous contaminants were detected in the reads that were generated from all three sequencing runs. Nearly all of the contigs assembled *de novo* from unmapped reads that were identified as representing a non-vertebrate species were comprised of reads that originated from multiple libraries sequenced at separate facilities. These attributes facilitated both the discovery and validation of the parasitic and commensal species sequences found in this study.

Despite the continuing exponential increases in sequences submitted to NCBI’s databases, the number of represented species still comprises only a small proportion of existing species. While we detected several sequence alignments to spirurid nematodes in both the DNA and RNA sequence data, none of these species are known to be present in the US or to infect taurine cattle. Therefore, we postulate that the actual species present within the tissues of Dominette either represent undiscovered species or a previously recognized, but unsequenced, organism such as *Onchocerca gutturosa* or *Onchocerca lienalis* from the Spirurida order. Both *O. gutturosa* and *O. lienalis* are known to infect taurine cattle in various parts of the United States [10]. However, these species have not been sequenced other than for a few selected genes used to generate data for phylogenetic analyses [11–19]. In this study, we assembled nearly 1000 contigs that we believe represent novel sequence from a Spirurid nematode that infects taurine cattle in North America.

The precise identity of the species generating the sequence matching *B. bigemina* in both the RNA-seq and genomic DNA data is also ambiguous. As no fever like symptoms were reported in this cow who spent her life at a USDA research facility near Miles City, Montana and babesiosis has been reported to have been eradicated in the United States with vaccination no longer being required [20], we suspect that Dominette was asymptomatically infected with a non-pathogenic strain of *Babesia* spp., as has previously been reported in Turkey [21], Syria [22] and Thailand [23]. Although it is currently not possible to determine the exact species of parasite, we can estimate the animal’s parasite burden via deep sequence data by evaluating the number of species to which the contigs of unmapped reads align and the number of contigs that align to each species. Parasite burden negatively impacts animal health and profitability [24, 25] and can serve as a reservoir for later infections [22]. Although symptoms were not visible and the animal appeared healthy, the detection of subclinical parasite burden, even from non-pathogenic parasites, is important because a physiological response to the infection from the host must still occur. This response reduces fitness, causes a decrease in production traits such as feed intake and feed efficiency [25, 26] and can also influence the interpretation of RNA-seq experiments.

An alternate explanation for the identification of non-vertebrate sequences in a vertebrate animal is the actual integration of these DNA sequences into the animal’s genome. Recently, horizontal gene transfer has been reported to occur at a low level in many animal species [27–29]. It has also been reported that there can be integration of foreign DNA released by dead cells into healthy host cells [30]. However, we were unable to find evidence for the integration of non-vertebrate DNA into this animal’s genome and must exclude horizontal gene transfer based on our data.

## Conclusions

In conclusion, we alert researchers that many sequences of interest may be found in the reads that fail to align to a reference assembly. We demonstrate that the unmapped reads contain biologically significant information relative to genes that are either partially or completely missing from the reference assembly, as well as information regarding the identity and magnitude of commensal or parasitic organisms. The large number of missing or misassembled bovine protein coding genes must significantly impact the interpretation of RNA-seq studies, warrants further research, and is likely more severe in the less complete reference genomes of other livestock species. Continuation of unmapped read mining will also expand our knowledge of the extent of internal parasitic infections and may lead to the discovery of previously unknown symbiotic relationships. These metagenomic inferences are an additional source of information from whole-genome sequencing data that can be used as phenotypes or covariates in downstream analyses. As the quality of reference assemblies improves and the scope of sequenced microorganisms broadens, the detection of parasitic infections and other symbiotic relationships will become more explicit.

## Methods

### Ethics statement

Tissues from L1 Dominette 01449 were sampled according to IACUC No. 081711–1, which was approved by the USDA-ARS Fort Keogh Livestock and Range Animal Care and Use Committee.

### DNA and RNA sequencing

DNA was extracted from liver and whole blood samples from L1 Dominette 01449 (referred to here as “Dominette”), a Hereford cow used to generate the *Bos taurus* Sanger reference assembly [7], and was sent to three separate facilities for sequencing. Paired-end and mate-pair libraries were constructed and DNA was 2 x 100 bp sequenced to an average coverage of approximately 55X. Further details regarding the sequencing of each library is provided in Additional file 1: Table S6.

RNA was extracted using Trizol Reagent (Invitrogen, Carlsbad, CA) as described elsewhere [31] from 17 tissue samples including ampula, blood, cerebral cortex, endometrium sampled from caruncular regions contralateral (car con) and ipsilateral (car ips) to the corpeus luteum, gallbladder, heart, ileum, infundibulum, jejunum, kidney, liver, mesenteric lymph nodes, pons, ribeye muscle, semitendinosus muscle, and spleen. Preparation of the mRNA samples for sequencing was performed by Global Biologics (Columbia, MO) using the TruSeq Stranded mRNA Library Prep Kit (Illumina®, San Diego, CA) and sequenced 2 x 100 bp using Illumina technology, with the exception of blood which used the TruSeq RNA Sample Preparation Kit and was sequenced 1 x 100 bp.

### Pre-processing and alignment of reads

Error correction was performed on DNA sequence reads using the QuorUM error correction algorithm [32]. After filtering duplicate and low quality reads, 1,622,097,087 unique reads remained. Paired reads were aligned to the UMD3.1 cattle reference assembly using NextGENe 2.4.1 (SoftGenetics, LLC, State College, PA) requiring at least 35 contiguous bases with ≥95.0 % overall match, up to 2 allowable mismatched bases, and up to 100 allowable alignments of equal probability genome-wide.

RNA sequence reads were filtered for quality and adapter sequences and were then trimmed using a custom Perl script already described [31]. Computations were performed on the HPC resources at the University of Missouri Bioinformatics Consortium (UMBC). TopHat v2.0.6 [33] was used to map the reads to the *Bos taurus* UMD3.1 reference genome. A total of 2 mismatches and up to 3 bp indels were allowed in alignment.

### De novo assembly of unmapped reads

Reads from DNA sequencing that remained unmapped following alignment to the reference genome were assembled using MaSuRCA 2.3.2 [34]. Reads from RNA sequencing that remained unmapped following alignment were assembled using Trinity version r20140717 [35]. For both DNA and RNA assemblies, the default parameters were used. To maintain a paired read file structure, reads where both the forward and reverse read were unmapped or where one of the reads was unmapped but the other was mapped were collectively used for assembly.

### Pairwise alignment of unmapped contigs to the nt database

Prior to pairwise alignment, contigs assembled from the unmapped DNA reads were sorted by size and only contigs greater than 500 bases were aligned (n = 42,086). Due to the smaller size of the RNA contigs, they were not filtered by size prior to pairwise alignment. Using the blastn algorithm of BLAST+ 2.2.30 [36, 37] and the command line options -db nt, -max_target_seqs 1, -outfmt "6 qseqid sseqid staxids sscinames pident length mismatch gapopen value”, each DNA and RNA contig was aligned to the NCBI non-redundant nucleotide database and the most significant alignment was returned. The -negative_gilist option was used with a text file of all *O. ochengi* gi numbers in subsequent blast searches excluding *O. ochengi* sequences. The BLAST output was then parsed to determine the subject species, percent identity, length of match, number of mismatches, number of gaps, E-value, and overall score. Significant alignments were declared only if the length of the alignment was ≥150 bp for DNA or ≥50 bp for RNA. Only the best match for each aligned contig was reported. This output was summarized according to the total number of alignments per species, mean, median, and maximum percent identity, mean, median, and maximum length of match, and mean and median E-value (Additional file 1: Tables S3 and S5).

### Quantification and identification of coding regions within unmapped reads

Contigs from unmapped RNA-seq reads were aligned to contigs from unmapped DNA reads using NextGENe 2.4.1 requiring ≥98 % overall match to declare a match. Additionally, for the significant RNA alignments, the gene symbol corresponding to the GI accession number for the alignment was captured where possible and recorded using the db2db tool in bioDBnet [38]. A unique list of gene symbols was constructed and the number of significant alignments to each gene was tallied (Additional file 1: Tables S7 and S8).

## Availability of data and materials

The data set supporting the results of this article is available in the SRA repository, SRA accessions SRX1177177 through SRX1177278.

## Additional files

Additional file 1:
**Table S1.** Statistics from the *de novo* assembly of unmapped reads from DNA sequencing. **Table S2.** Statistics from the *de novo* assembly of unmapped reads from RNA sequencing. **Table S3.** Summary of all significant alignments from pairwise alignment of *de novo* assembled contigs from DNA unmapped reads to the *nt* database. **Table S4.** Number of significant alignments per tissue from pairwise alignment of *de novo* assembled contigs from unmapped RNA-seq reads to the *nt* database. **Table S5.** Summary of all significant alignments from pairwise alignment of *de novo* assembled contigs from unmapped RNA-seq reads to the nt database. **Table S6.** DNA sequencing metadata. **Table S7.** Genes represented in alignments of *de novo* assembled contigs from unmapped RNA-seq reads to *Bos taurus*. **Table S8.** Genes represented in alignments of *de novo* assembled contigs from unmapped RNA-seq reads to *Bison bison bison*, *Bubalus bubalis*, or *Bos mutus*. (XLSX 731 kb) Additional file 2:
**Note 1:** The *Onchocerca ochengi* reference assembly is contaminated with bovine genomic sequence. **Note 2:** Estimation of the number of protein coding genes missing or misassembled in the UMD3.1 bovine reference assembly. (DOCX 41 kb)
