# Supplementary material for: What’s in your next-generation sequence data? An exploration of unmapped DNA and RNA sequence reads from the bovine reference individual
Source: BMC Genomics. 2015 Dec 29;16:1114. doi: 10.1186/s12864-015-2313-7 (PMC4696311; doi:10.1186/s12864-015-2313-7)
Supplement: Additional file 2: — Note 1: The Onchocerca ochengi reference assembly is contaminated with bovine genomic sequence. Note 2: Estimation of the number of protein coding genes missing or misassembled in the UMD3.1 bovine reference assembly. (DOCX 41 kb) [file 12864_2015_2313_MOESM2_ESM.docx]

**Supplementary Note 1: The *Onchocerca ochengi* reference assembly is contaminated with bovine genomic sequence.**

After detecting a significant number of alignments to *O. ochengi* and *B. bigemina*, both known to be bovine endosymbionts, we generated pseudo paired-end read data from the *O. ochengi* (GenBank accession number GCA_000950515.1) and *B. bigemina* (GenBank accession number GCA_000723445.1) reference assemblies deposited in NCBI’s assembly database. One hundred bp paired-end reads were generated with a random library DNA fragment size distribution between 200 and 500 bases (mean of 350 bases) by stepping through the genome in 1 bp steps. A total of 100X coverage of each genome was simulated. The same procedure was used to generate pseudo reads for five other genomes that were not detected in the unmapped read contigs and also for *O. volvulus*, the human counterpart of *O. ochengi* (Table 1; GCA_000499405.1, GCA_000001215.4, GCA_000002985.3, GCA_000469785.1, GCA_000612645.1, and GCA_000816705.1).

**Table 1**. Alignment statistics of simulated paired-end read data to the UMD3.1 reference.

|  | **Total reads** | **Unmapped reads** | **Mapped reads** | **Percent mapped** |
| --- | --- | --- | --- | --- |
| *Babesia bigemina** | 27,006,638 | 26,992,174 | 14,464 | 0.0536% |
| *Onchocerca ochengi** | 157,944,910 | 155,336,678 | 2,608,232 | 1.6514% |
| *Onchocerca volvulus* | 191,873,454 | 191,867,012 | 6,442 | 0.0034% |
| *Drosophila melanogaster* | 284,838,326 | 284,818,193 | 20,133 | 0.0071% |
| *Caenorhabditis elegans* | 200,536,826 | 200,532,573 | 4,253 | 0.0021% |
| *Echinococcus granulosus* | 221,623,539 | 221,527,755 | 95,784 | 0.0432% |
| *Trichuris muris* | 166,474,643 | 166,344,036 | 130,607 | 0.0785% |
| *Dictyocaulus viviparus* | 311,922,279 | 311,368,169 | 554,110 | 0.1776% |
| *Species detected in resequencing | |  |  |  |

The alignment of the *O. ochengi* pseudo reads to the bovine reference genome produced significantly more mapped reads than did the alignments for the other species, including *O. volvulus*, a very closely related species (Table 1). Read mapping rates were significantly different (*Χ^2^*= 16,714,393, df = 7, p-value ≈ 0) and read mapping was significantly different for *O. ochengi* (*Χ^2^* = 16,417,090, df = 1, p-value ≈ 0). Less than 0.004% of the *O. ochengi* reads that were aligned to the cattle reference assembly could be aligned to *O. volvulus*, while over 97% of the *O. ochengi* reads that did not align to the cattle assembly could be aligned to *O. volvulus*. Furthermore, when the aligned reads were individually aligned to the *nt* database the most significant alignment was always to *O. ochengi*, but all other significant alignments were to *Bos taurus* or other mammals. The *O. ochengi* sample originated from cattle skin, and when we downloaded the raw FASTQ files used to generate this assembly and aligned them to the cow reference, over 33% of the reads aligned. While the majority of the cattle sequences were filtered from the dataset, the *O. ochengi* genome assembly contains contamination from cattle sequence. Therefore, we excluded this genome from further BLAST searches because we could not distinguish alignments to this genome being due to the presence of nematode sequence in our data or cattle sequence in the nematode assembly. This resulted in an increase in the number of alignments to other nematodes from the order Spirurida. All species that were subsequently detected are only known to infect humans. Thus, they were presumably detected due to the presence of a genetically similar, but unsequenced, nematode(s) in our sample.

**Supplementary Note 2: Estimation of the number of protein coding genes missing or misassembled in the UMD3.1 bovine reference assembly.**

To arrive at an estimate of the quantity and types of genes that may be missing or misassembled in the UMD3.1 reference assembly, we mapped the GI number from the most significant BLAST alignment of each RNA contig to a gene symbol. Based on this mapping, we estimate that 4,412 annotated bovine genes were represented in the 17,856 alignments to *Bos taurus* sequences. A complete list of the affected genes can be found in Table S7. Of the 13,769 significant alignments to bison (*Bison bison bison*), water buffalo (*Bubalis bubalus*), or yak (*Bos mutus*), 4,029 genes were represented. Only one gene was found in common between the 4,412 detected from alignments to *Bos taurus* sequences and the 4,029 detected from alignments to bison, water buffalo, or yak. A complete list of these genes is presented in Table S8. We also aligned the RNA-seq contigs to our set of DNA contigs and found that approximately 21% aligned with greater than 98% identity. Of the 46,690,692 bases represented in the DNA contigs, the RNA contigs covered 1,232,501 bases. This is equivalent to 2.6% of the bases which is slightly greater than the genome-wide estimate of coding regions, which has been estimated to be less than 2% of the genome in mammals [1, 2].

Based on these analyses, it is apparent that there is a large amount of coding sequence that is either absent or misassembled in the UMD3.1 bovine reference assembly, resulting in the matching of these unmapped contigs to other vertebrate or *Bos taurus* sequences. The extent to which the bovine coding sequences are affected by misassembly is difficult to determine. Florea *et al.* indicated that the UMD3 assembly, although generally superior, does not contain 660 genes that are present in UMD2 [3]. We detected significant alignments to 8,440 unique genes suggesting that as much as 42% of the bovine protein coding genes (http://useast.ensembl.org/Bos_taurus/Info/Annotation?redirect=no) are misassembled*.* In terms of total nucleotides, we assembled approximately 9.5 million bases of unmapped reads from RNA sequencing data that aligned to cattle, bison, water buffalo, or yak. Given that the coding regions represent around 2% of the genome, the total number of coding nucleotides is approximately 53 million.

Based on the lengths of the transcripts assembled (median N50 of 334 bp across tissues), we predict that many of these may be minor misassemblies, such as a missing exon. However, transcripts as large as 6,591 bp were also assembled from the unmapped reads, indicating that there are large portions or even entire genes missing as well. Clearly, improvement of the bovine reference assembly is essential to correctly interpret genome resequencing and RNA-seq data and maximize progress from sequencing efforts. This conclusion has now been reached by several research efforts [4–9], and new long read sequencing technology will likely play a significant role in reference assembly improvement [10].

**References**

1. Lander ES, Linton LM, Birren B, Nusbaum C, Zody MC, Baldwin J, Devon K, Dewar K, Doyle M, FitzHugh W, Funke R, Gage D, Harris K, Heaford A, Howland J, Kann L, Lehoczky J, LeVine R, McEwan P, McKernan K, Meldrim J, Mesirov JP, Miranda C, Morris W, Naylor J, Raymond C, Rosetti M, Santos R, Sheridan A, Sougnez C, et al.: Initial sequencing and analysis of the human genome. *Nature* 2001, 409:860–921.

2. Venter JC, Adams MD, Myers EW, Li PW, Mural RJ, Sutton GG, Smith HO, Yandell M, Evans CA, Holt RA, Gocayne JD, Amanatides P, Ballew RM, Huson DH, Wortman JR, Zhang Q, Kodira CD, Zheng XH, Chen L, Skupski M, Subramanian G, Thomas PD, Zhang J, Gabor Miklos GL, Nelson C, Broder S, Clark AG, Nadeau J, McKusick VA, Zinder N, et al.: The sequence of the human genome. *Science* 2001, 291:1304–51.

3. Florea L, Souvorov A, Kalbfleisch TS, Salzberg SL: Genome assembly has a major impact on gene content: a comparison of annotation in two Bos taurus assemblies. *PLoS One* 2011, 6:e21400.

4. Kelley DR, Salzberg SL: Detection and correction of false segmental duplications caused by genome mis-assembly. *Genome Biol* 2010, 11:R28.

5. Zimin A V, Kelley DR, Roberts M, Marçais G, Salzberg SL, Yorke JA: Mis-assembled “segmental duplications” in two versions of the Bos taurus genome. *PLoS One* 2012, 7:e42680.

6. Salzberg SL, Yorke JA: Beware of mis-assembled genomes. *Bioinformatics* 2005, 21:4320–1.

7. Church DM, Schneider VA, Graves T, Auger K, Cunningham F, Bouk N, Chen H-C, Agarwala R, McLaren WM, Ritchie GRS, Albracht D, Kremitzki M, Rock S, Kotkiewicz H, Kremitzki C, Wollam A, Trani L, Fulton L, Fulton R, Matthews L, Whitehead S, Chow W, Torrance J, Dunn M, Harden G, Threadgold G, Wood J, Collins J, Heath P, Griffiths G, et al.: Modernizing reference genome assemblies. *PLoS Biol* 2011, 9:e1001091.

8. Church DM, Schneider VA, Steinberg KM, Schatz MC, Quinlan AR, Chin C-S, Kitts PA, Aken B, Marth GT, Hoffman MM, Herrero J, Mendoza MLZ, Durbin R, Flicek P: Extending reference assembly models. *Genome Biol* 2015, 16:13.

9. Genovese G, Handsaker RE, Li H, Kenny EE, McCarroll SA: Mapping the human reference genome’s missing sequence by three-way admixture in Latino genomes. *Am J Hum Genet* 2013, 93:411–21.

10. English AC, Richards S, Han Y, Wang M, Vee V, Qu J, Qin X, Muzny DM, Reid JG, Worley KC, Gibbs RA: Mind the gap: upgrading genomes with Pacific Biosciences RS long-read sequencing technology. *PLoS One* 2012, 7:e47768.
